# Electronic Design for Wearables Devices Addressed from a Gender Perspective: Cross-Influences and a Methodological Proposal

**DOI:** 10.3390/s23125483

**Published:** 2023-06-10

**Authors:** Elena Romero-Perales, Clara Sainz-de-Baranda Andujar, Celia López-Ongil

**Affiliations:** 1 Departamento de Tecnología Electrónica, Universidad Carlos III Madrid, 28911 Leganés, Spain; celia@ing.uc3m.es; 2 Instituto de Estudios de Género, Universidad Carlos III Madrid, 28903 Getafe, Spain; cbaranda@hum.uc3m.es; 3 Departamento de Comunicación, Universidad Carlos III Madrid, 28903 Getafe, Spain

**Keywords:** wearables, gender, electronics design, methodology, multidisciplinary, user center design

## Abstract

The design of wearable devices has been approached from many perspectives over the years, mainly from a functionality, electronics, mechanics, usability, wearability, or product design perspective. However, there is a missing point in these approaches: the gender perspective. Gender intersects with every approach and, considering the interrelationships and dependencies, can achieve a better adherence, reach a wider audience, and even change the conception of the wearables design paradigm. The electronics design addressed from a gender perspective must consider both the morphological and anatomical impacts and those emanating from socialization. This paper presents an analysis of the different factors to consider when designing the electronics of a wearable device, including the functionality to implement, sensors, communications, or the location, together with their interdependencies, and proposes a user-centered methodology that contemplates a gender perspective at every stage. Finally, we present a use case that validates the proposed methodology in a real design of a wearable device for the prevention of gender-based violence cases. For the application of the methodology, 59 experts have been interviewed, 300 verbatims have been extracted and analyzed, a dataset from the data of 100 women has been created and the wearable devices have been tested for a week by 15 users. The electronics design needs to be addressed from a multidisciplinary approach, by rethinking the decisions taken for granted and analyzing the implications and interrelationships from a gender perspective. We need to enroll more diverse people at every design stage and include gender as one of the variables to study.

## 1. Introduction

The design of wearable devices has been gaining importance during the last decade. These devices are widely used for many applications such as activity tracking, health monitoring, sports practice, interaction with other devices, sleep quality, contextual enhancement, or security [1]. 

Wearable devices are electronic devices designed to fulfill a functionality characterized by the users wearing them on their bodies. This fact implies considering anatomical and physiological factors, since there are morphological differences between men and women. However, the design with a gender perspective should not stop there, since it is also necessary to assess the behavioral and sociological differences between genders, because the final design will have a different impact on them. The design of electronic devices can reveal unconscious biases and negatively impact the functionality, behavior and user experience for women and non-conforming gender persons. 

In this work, we use a gender approach for sex and gender. Sex refers to the biological concept that includes factors such as genes, hormones, chromosomes, anatomy and physiology, whereas gender is a complex human sociocultural and environmental concept [2]. Like sex, gender is often misconceptualized and misrepresented as a binary construct (i.e., men/women), but there are many more variations. In any case, both anatomical and sociocultural characteristics must be considered throughout the design cycle if we want to design for cis men, women, transgender and gender non-conforming people.

Androcentrism [3] refers to the practice of center culture, society, economics, politics and science around men and their characteristics, needs, priorities and values, while ostracizing women to the periphery. Androcentrism also refers to men as the gender-neutral standard, defining women as specific or “the other” [4]. 

Science, research and electronics design (like many other areas) are no more free of androcentrism. Women have been ignored as a target group regarding the choices and design of technology when they have not been subject to gender stereotyping in terms of the use of technology and products [5]. This perspective affects both the functionality provided by the devices (addressed for the male needs), the design, shape and location (for male body anatomy), as well as their daily routines (patterns associated with the masculine).

This problem does not only apply to the design of electronic devices, but sometimes tends to be invisible in science and technology, considered objective and neutral areas. Usually, when a device is designed as genderless, it promotes the masculinist ideal, forgetting about gendered issues and concerns [6]. Self-reflection and feminism are needed to improve science. We need to rethink the role of technology related to gender [7].

Taking into account the gender perspective in the design of products in general and electronic devices and wearables in particular is a crucial aspect for several reasons. First, it is social justice (to compensate for years of not taking into account women in design), second, because women make up 50% of the population and are therefore equally likely users, and finally, because it increases the adherence to wearables for existing applications and devices, and more importantly, addressing the design from a feminist perspective would open a range of currently unknown new designs, devices and features to be covered.

In the field of wearable device research, some works focus on the analysis of the functionalities they can offer, the aesthetic, shape and materials of the devices, how they affect relationships and social roles security, privacy and the protection of collected data. Some of these studies, which come from social sciences (sociology, anthropology, law, ethics), approach the influence of wearable devices and how they affect privacy, sociology, rights, or human interactions from an anthropological point of view. In these areas, the gender perspective is sometimes present, and we can find analyses from a feminist perspective that analyze the usability, acceptance, or behavioral changes from this point of view [8,9,10]. 

If we approach the research on electronic device design from the engineering point of view, the contributions to science focus on the functionality to cover, the device’s location and ergonomics, the sensors included, the power supply methods, communication protocols, the computing paradigms, or user interfaces [1,11,12]. 

We advocate that the design process involving electronics (usually assumed to be neutral) needs to be addressed from a gender perspective as well. In this work, we focus on the gender perspective and scrutinize every aspect of the design of wearable electronic devices that is affected or affects the gender aspects. If we want to design inclusive devices, we should also incorporate sexual orientations, and racial, functional, and age diversity. Further studies must address this topic from an intersectional approach.

We also advocate an interdisciplinary approach, since gender perspective analyses performed from the social area are not impermeable and should work hand in hand with the electronics design itself in an interdisciplinary co-design methodology [13].

Among the previous work in the approach of incorporating a gender perspective in the design of electronic devices, there are some works that analyze the usability, comfort, wearability and acceptance of different devices and applications according to gender, but these analyses are closer to the field of design and marketing than engineering. We need a global approach, since other elements taken for granted (sensors, communication technologies, battery life, etc.) should be rethought, and then they will affect the overall system design. Every aspect impacts on others and we need to address it systematically.

For that purpose, the main contributions of this work are:

- An analysis of the different aspects to consider in the design of a wearable device and the implications they have in developing an inclusive design from a gender point of view.
- A proposal of a design methodology that incorporates the gender perspective from the electronics point of view for a wearable device. We also analyze how they affect each other and the intra-implications and the cross-implications.
- A use case of application of the methodology presented for a wearable device design to protect gender-based violence victims.

The rest of the paper is organized as follows: Section 2 covers the analysis of the different elements included in the wearable design addressed by gender and how every aspect has gender influence, along with the concepts to consider when deciding about every module. Section 3 presents the methodology proposed to systematically address the wearable design considering gender together with the interdependencies affecting every block. Section 4 uses the previously presented methodology and applies it to the design of a wearable intended to protect victims of gender-based violence. Finally, Section 5 presents the conclusion of this work.

## 2. Analysis for Gender Perspective Inclusion

**Figure 1:**
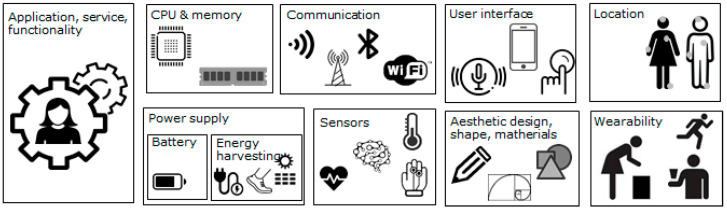
Elements of a wearable device.

A wearable device consists of the following main elements from a hardware electronics perspective: a processing unit, memory, sensors, a radio transceiver, and a power supply. Apart from paying attention regarding the gender perspective in the selection, design and configuration of these blocks, there are other important considerations to analyze included in the design process. These are the implemented functionality itself, the location of the device, the material and shape of the device or its encapsulation, the user interface and the software that manages its operation. In every one of the aforementioned components, incorporating a gender perspective in their selection, design and integration is fundamental and we will discuss them in depth in the following subsections following the elements depicted in Figure 1.

### 2.1. Functionality and Application

Firstly, and before beginning the design process itself, the final functionality and the wearable requirements should be defined with people’s needs and desires (where people include a variety of different people in terms of gender, age, race, sexuality, class, body types and functional abilities) as the main element, instead of just creating a new wearable product for the market. The users must be involved in the design process and their needs and requirements must be a central part of the procedure. Traditionally, the prototype of the user and designer is mainly a young white man within the information and communication technology world, which implies an important set of biases in all the design stages, decisions and results. Eliminating these biases requires their in-depth knowledge, as well as the conscious question about technology’s role for society. The solution is not only recruiting women for wearable design teams, but also and mainly to change the perspective about technology, devices, needs, interactions and the analysis of the consequences [14,15].

Applying a gender perspective in the design process also means opening the focus and paying attention to different possible applications and interests instead of continuing to delve into wearables mostly focused on sports and productivity enhancement, and looking toward other service models and applications. This is not because women are not interested in these functionalities, but because other options can be explored, attending to the demands of other groups, thus enriching the quality of life of all. 

Moreover, it is important to pay special attention to the problem of reinforcing gender stereotypes through the functionalities of wearables, about how being a woman is understood and what is expected of them [9]. Applications such as geolocation trackers designed to increase security replicate and deepen the idea that women in public spaces are in danger and that they should be protected. Another problem is the potential misuse of wearable devices when gender conditions are not considered and data privacy is not guaranteed. For example, promoting or increasing eating disorders through fitness trackers [6], the use of location devices by ex-partners to harass and control, or recently, the risk of using information from period trackers to persecute and accuse women of seeking or considering an abortion.

In terms of functionality, one of the issues that have been attracting more research in terms of a gender perspective for some years now is the biases of artificial intelligence algorithms. These biases operate not only in terms of gender, but also in terms of racial diversity, class, or functional diversity. As stated in [16], two of the fundamental problems are the database used for training (unbalanced and misrepresented) and the bias in the algorithm itself that should be curated. As a first approach, the training data must include diverse people so that the algorithms do not learn to identify or classify exclusively white male features; however, this may not be sufficient. It is necessary to analyze the behavior of the algorithm and inspect if it operates differently and the reason in order to subvert it. Finally, the devices used to collect the data for training must also be taken into account. On the one hand, if the data are obtained from wearable devices currently in use, the bias will be perpetuated, since those who already have a wearable device belong to a certain profile (probably male, white, and with a medium–high class). On the other hand, if the data are collected with a device designed without a gender perspective, the design itself will introduce bias in the behavior and the use of the wearable. 

Furthermore, the validation tests on end-users should not be postponed till the final stages in the design cycle, but include these users from the beginning, not only for testing, but also during the product definition. Therefore, not only the definition of the problem to solve, but also the functionality, use cases, context, requirements, etc., will benefit from a multidisciplinary and broad approach. For example, if we only analyze the usage rates of a device for assessing its success, the results probably cannot be generalizable if the device had been designed considering only a portion of the population, and therefore the lack of use for other populations groups is not meaning lack of interest but unsuitability.

### 2.2. Sensors

In wearable devices, sensors are required to monitor the user’s activity and/or health/wellness parameters. Typical wearable devices (such as activity bracelets, glasses/headsets, jewelry, clothing and implantable medical devices) usually have sensors to monitor magnitudes such as temperature, sweating, heart rate, cardiac activity, brain activity, muscle activity, respiration, movement and the situation around the user. The signals provided by these sensors will be crucial to accurately execute the device’s functionality. Nowadays, there are a wide variety of smart sensors that can measure physical or physiological data with a high degree of quality. 

As the number and diversity of sensors to include are quite large, we are going to focus on analyzing those related to human characteristics (such as temperature, pulse, cardiac and brain activity), as the influence of gender is even more profound due to anatomical differences apart from those related to social behavior. Additionally, a similar analysis should be performed regarding gender for the use of accelerometers, cameras, or location sensors. 

During the design process of the wearable device, two factors should be considered concerning sensors and gender. First, the choice of the variable to be monitored (which variables are worth for the final application and what is the best way to measure them), and second, if the variables and sensors chosen have different ranges or behaviors depending on the population considered, in this case, if they present gender differences.

Within the first aspect, sensors selected for a specific application could be different depending on gender. For example, the most significant magnitudes and characteristics used for predicting thermal comfort depend on gender according to [17]. In [18], different features chosen for detecting heart rate variability show different results depending on gender. Addressing this situation from a gender perspective needs to collect, analyze, and depict the results in a disaggregated manner to be able to conclude which are the most influential signals or features for the classification/detection of different states or conditions (environmental, emotional, physical activity, etc.) according to the users’ gender.

In the second aspect, and bearing in mind the magnitudes to be measured, listed above, we must consider the differences in these variables concerning gender. Previous research shows differences in the behavior of physiological variables such as temperature, sweating, heart rate, electrocardiogram (ECG), electroencephalogram (EEG), or electromyogram (EMG), which we will detail in the following sections according to gender. These differences occur both in the usual ranges of measurement and in the behavior of the different variables depending on the situation.

A very important aspect in the design of wearables and the decision of which sensors to include is their accuracy and their metrological performance. In this regard, we can find works that review the existing methods for measuring accuracy and provide guidelines for the correct measurement of the sensors [19]. Gender should be included as one of the variables to be taken into account when measuring whether the developed sensors perform satisfactorily for the end-user population. In this work, an emphasis is placed on collecting and providing data from test subjects so that conclusions can be drawn.

#### 2.2.1. Temperature Sensor

Temperature sensors are usually included in wearables due to their low cost for multiple applications. They could be used in health, emotion, or activity trackers, as well as those used for work environment safety. 

There are gender differences in the absolute body temperature, the distribution of body temperature, and its variation in different situations. As portrayed in [20], women’s body temperature is usually lower than men’s, especially if we compare the body temperature of the thighs and trunk. Likewise, significant differences are described in [21] for most body parts, especially in the legs (thighs and shank), and practically no difference in the hands. 

Women thermoregulate differently in response to sudden changes in temperature [22], showing a larger range of body temperatures. When faced with different temperature scenarios [23], differences (lower body temperatures) are also found according to gender. Therefore, these variations should be taken into account, as they may affect the design of a wearable used for detecting emotional states, monitoring sports activity, or helping in the detection of diseases. It is necessary to advance in research and do so from a gender perspective.

In addition, there are differences in body temperature throughout the menstrual cycle [24] that may need to be considered when designing some systems, together with hormone therapies [25] or pregnancy [26].

Not only the body temperature is different depending on gender, but also the influence of the different magnitudes for achieving thermal comfort. According to [17], the models for predicting thermal status should be designed differently for each gender group. Not only are the different temperature ranges considered optimal for men and women, but also the features used in the model for determining which temperature is optimal for men and women are different. 

#### 2.2.2. Heart Rate Sensor

Heart rate sensors are quite common in activity bracelets for health monitoring and for those used to measure physical activity and fitness improvement [27]. From this measurement, it is easy to extract the heart rate, oxygen concentration and other parameters related to heart activity. Blood volume pulse (BVP) sensors are based on photoplethysmography (PPG) using light reflection and refraction through the skin and superficial blood vessels. Light-emitting diodes (LED), with different light colors and photodetectors, are commonly used in these sensors to implement the PPG measurement technique. Optical sensors are cheap and easy to integrate into wearables, but they present disadvantages due to their high-power consumption and low accuracy when located on the wrist area. Motion artifacts, skin types, signal crossover and even tattoos greatly affect BVP sensors, provoking a lack of accuracy and high computing effort in data filtering and quality recovery algorithms [28] ). As stated in [29], chest straps present better results in terms of accuracy. However, heart rate also shows variations according to gender that should be taken into account. 

According to [18], differences are found in both the heart rate (HR) (higher in women) and heart rate variability (HRV) (in this case, lower in women). In addition, these differences also depend on age, being more pronounced in young women. This study also shows that different results are obtained depending on the features chosen for detecting heart rate variability, thus confirming the hypothesis that the choice of the features to monitor is also determined according to and dependent on gender. In [30], the authors stated the slight differences in HRV for young women in some of the features analyzed.

Differences are found not only in ordinary situations, but also under stress, exercise, or different emotional states. According to [31], HRV analyzed in situations of prolonged states of sadness for 24 h increases in women and decreases in men. Under stressful situations, the heart rate varies, increasing even more in women [32].

In addition, since motion is one of the most influential factors for artifacts in the measurement [33], we should ask ourselves whether the daily activities influence the measurement depending on gender. Do the routines of men/women/gender non-conforming people include different types of movements? 

#### 2.2.3. Galvanic Skin Response (GSR)

Galvanic skin response is commonly measured to identify different activation states (mainly stress) in persons, commonly employed for fitness or emotional trackers. The automatic response of the sympathetic nervous system (SNS) to stimulus reception provokes changes in the skin glands related to sweating activity [34]. GSR is measured as the electrical conductivity of the skin, which increases with the perspiration and presence of sweat on the skin surface. The variation in the galvanic skin response is a low-frequency signal with two main components: phasic and tonic.

As in the case of temperature, gender differences are also found in the galvanic skin response. As stated in [35], men sweat much more than women in similar situations. Sweating plays a major role in the thermoregulation process too, so it is affected by gender, as described in the temperature section. Moreover, the authors document in [36] significant differences in sweating under heat stress as a function of gender due to physiological differences, even when eliminating confounding variables. This aspect needs to be further explored to see the influence of gender in different emotional or stressful situations. 

Similarly, as in the case of temperature, sweating is also affected by hormonal cycles and menopause, which should be considered when designing the system and when customizing the final application. It is not just a problem of bias, but sensitivity and signal ranges. Furthermore, in the case of measuring the GSR, the position, size, and material of electrodes are key factors to consider and study regarding gender responses.

#### 2.2.4. Electrocardiogram (ECG)

Electrocardiography sensors are quite commonly included in wearables, primarily associated with medical applications, although lately also in fitness or emotional trackers.

An electrocardiogram sensor measures the electrical activity of the heart. It measures the electrical potential difference between several points. Sensing the voltage difference requires the placement of electrodes on the body located at specific points. The most common electrocardiograms used in medicine measure 12 voltage differences (leads) from 10 electrodes, located on the trunk, legs and arms.

In wearables devices, to increase their wearability and comfort, fewer leads (1 or 2) are usually measured. Common shapes and locations for ECG sensors are embedded in clothes (such as bras), chest patches, chest straps, or smartwatches. In the case of measuring ECG through the fingers, differences in thickness and conductivity of the skin should also be considered.

As in the case of HR (many references are shared), there are documented differences in the electrocardiogram signal (ECG) according to gender. ECG sensor inaccuracies are more delicate since they are more frequently integrated into medical monitoring devices. 

In particular, regarding the inclusion of ECG in wearable devices, according to the review [37], only one of the studies reviewed reported the results as a function of gender, so they encourage further research in this topic and follow-up on the results, since the data are scarce and do not allow for a conclusion to be made. Even the study that addresses gender as one of the variables [38] points to its limitations regarding the sample size, which should be expanded to deepen the results. 

In addition, the signal artifact ratio (SAR) is described in this study [39] as better in men than in women without specifying the cause. It should be important to assess whether part of this worse performance is due to the design of the system itself (material, technique, location of the sensors, etc.) because of androcentrism.

Apart from the wearables, but also for situations in which they are used commonly, such as emotion detection, sports performance and medical monitoring, there are differences in ECG measurements according to gender, for example, in the face of aggression stimuli [40], or for athlete performance [41], where there are differences not only in the HR, but also in other ECG features.

#### 2.2.5. Electroencephalogram (EEG)

An electroencephalogram (EEG) sensor measures the electrical activity in the brain. This measure has a high temporal resolution, where even the spatial resolution is a disadvantage of this method. An EEG sensor measures the electrical potential difference between several points. As for the ECG, it is necessary to place the electrodes on the body (in this case on the scalp) at specific points. Usually, the international 10–20 system is employed for addressing the electrodes used. There are many different placements for the electrodes in an EEG depending on the purpose of the measure, usually ranging from 25 to 64 electrodes for medical, research, or ambulatory uses [42]. Obviously, for wearable sensing, the number of electrodes is required to be reduced to increase the wearability of the device. The number of electrodes ranges from 2 to 14, usually located in the frontal area.

Although an electroencephalography sensor is not the most common sensor in wearable devices, it is true that lately, the number of devices used to measure brain activity for improving tasks such as meditation, emotional awareness, concentration, relaxation, or attention in educational or wellness applications has increased. 

For emotional response, differences are reported both in the spectrum power for all frequency bands and spatial distribution (especially for negative emotions) for men and women [43]. Regarding relaxation, concentration, or sleep quality monitoring systems, in [44], so many differences between genders are identified both in spectral power and in different oscillatory activities for deep sleep states, that the conclusion states that for this type of study, gender should always be taken into account. In [45], a broader study covering different tasks (eyes open/closed, relaxation, video viewing, and connection tasks) and differences in spectral power are also reported for all cases, reaching the same conclusion.

#### 2.2.6. Electromyogram (EMG)

Electromyography (EMG) is the recording of the electrical activity of the muscles. As in the previous cases (for ECG and EEG), we need a pair of electrodes to measure the difference in voltage generated by muscle activity. The placement of these electrodes will depend on the muscle of interest for the final application. 

Regarding the use of EMG sensors in wearable devices (mainly used in fitness applications and the optimization of sports practice), there are differences in muscle response and muscle contractions/distensions to different movements. Thus, in [46], they refer to the differences in extension amplitude, as well as variations in inter-muscular patterns that could explain different types of neck/shoulder injuries for repetitive movements.

Apart from the differences found regarding physical activity, there is another source of dissimilarity related to emotional expression, mainly facial muscle activation. In [47], the authors reported differences in facial reactions in approval situations. According to [48], women report a more pronounced facial expression for the expression of emotions. However, it is necessary to continue research in this area, since the studies cited above compare the emotional response to different stimuli, but lack information for the measurement system or sensor location and their differences regarding gender.

#### 2.2.7. Respiration

Previous works on differences in breathing patterns are scarce in the case of wearable devices. Regarding the applications covered by wearable devices to date, differences in breathing-related difficulties during panic attacks [49] are found, while according to [50], no differences are found between men and women in breathing patterns for tasks with a high metabolic demand as a function of activity (fitness, spinning, etc.). Related to sensor selection, the authors in [51] review possible gender differences in the use of a PPG sensor to measure respiration, concluding that there is no significant difference.

Regarding respiration in general, regardless of whether the devices are wearables, several works compile physiological and social differences in the respiratory system, reporting gender differences that occur throughout a person’s lifespan, from birth to old age, also dependent on the menstrual cycle, hormone treatments and menopause [52]. However, further research is needed, since sometimes, the sex variable and its relationships with the size of the different organs can be misinterpreted. 

Table 1 summarizes the identified differences for every physiological signal. However, further studies are needed and encouraged to deepen the knowledge and collect data that could state and explain the differences, if found. 

### 2.3. Central Processing Unit (CPU) and Memory

CPU and the memory are the core parts of the wearable device and are responsible for managing the different tasks and processes, monitoring and processing sensor signals and handling the user interface (if integrated into the wearable). Most wearable devices use a microprocessor, a microcontroller, or a System on Chip (SoC) that integrates the microcontroller and the communication transceiver. They usually have the memory integrated, but it is also possible to add external memory to the device. 

The selection of the core processing unit and the memory generally depends on the processing capabilities needed for the wearable (some final applications, sensors, or user interfaces require heavy computing, while others are quite low-demanding in terms of processing capabilities). For wearable devices, the most significant decision regarding CPU and memory selection is whether to process data on the wearable (processing on the edge) or send the information to be processed on other devices (fog or cloud computing). Some wearable devices perform complex algorithms for sensor processing or machine learning that could be implemented in the device itself (requiring higher processing capabilities) or on other devices (requiring higher data transmission). This trade-off is one of the key points for the selection of this module. In addition to the influence on communications, the power supply is also affected by this decision.

Regarding the gender approach, neither morphophysiology nor gender socialization affect the selection of the specific microcontroller or memory system, but this gender perspective could affect the selection of the type of computing (edge vs. fog/cloud) because this decision may depend on the availability of communications, the power supply, or the user interface to be used, which will impact the selection of the CPU and memory. These topics will be discussed in the following sections.

### 2.4. Power Supply

When designing a wearable system, the power supply is also a crucial issue. The device must move with the wearer, must and therefore be powered by batteries. The weight and size of the batteries are fundamental for the acceptance of the final device. This aspect has a lot to do with the location and final design of the device, since the weight or size will be determined not only by the functionality, but also by its location.

A state-of-the-art power supply using batteries for wearable devices is oriented at finding new technologies, increasing power density, flexible materials, or lengthening the duration of the same. However, no work addresses the choice of batteries from a gender perspective. Are the materials used better suited in some scenarios than others? In regards to the technology used, are some easier to embed in some accessories or garments? When embedding wearable devices in clothing (of which the material, durability and shape differ depending on gender), are there options that better suit the different materials or clothing? We cannot forget that the size of the battery is often the bottleneck for reducing the final size, shape and weight of the device.

The battery duration chosen as the standard is often adjusted to behavioral patterns: Do we think about these patterns from an androcentric point of view? Is the time working indoors or the use of public/private transport and the time and availability to charge the device taken into account? Total battery discharge is also a critical factor in some applications. In general, it is never desirable for the battery to run out, but what happens when people’s lives are at risk [53]?

Some of these devices also use energy harvesting (EH) techniques for recharging batteries during operation [54], and some of these techniques are related to the context and use conditions of the system (solar, wireless RF) or the user (thermal, piezoelectric) [55]. We should consider that the context, situation, movement patterns, daily activities and routines, and physiological conditions depend on gender. According to the user’s movement, hours of sun exposure and availability of radiofrequency radiation in the proper band, should the EH method choice depend on gender? Furthermore, there are differences in average weight and body temperature, as we have stated before, that impact piezoelectric or thermal EH methods. 

Beyond the user’s preferences, it is also necessary to test these EH techniques on different people with different routines (because the persona used for testing and validating the devices, again, is a young white male, without functional diversity and usually working in a research laboratory). As in all previous cases, it is necessary to broaden the sample. 

Battery maintenance and recharging [56] is a common annoyance when mentioning causes for low adherence to wearable use together with not fitting the user’s routines. In [57], the authors studied the patterns of use and battery charging in wearable devices; however, their sample only had two women versus 57 men for the online survey (being unbalanced, misrepresenting females), and there are no data regarding the sample for the in-depth battery usage analysis presented. The results establish that the battery status is checked less for smartwatches than for smartphones and that users tend to recharge wearables at night. Additionally, one of the drawbacks is the exclusivity of the charger. Without results disaggregated by gender, we cannot draw any conclusions.

The chosen power supply, battery life, EH methods used, frequency, time, and type of charging method should be considered when designing the wearable device, and of course, studying these factors from a gender perspective.

### 2.5. Communication

Communications (usually wireless) play a fundamental role in the design of the wearable system. Although wearable devices commonly use widely common technologies such as Wi-Fi and Bluetooth, other devices use ad hoc communication protocols or even communication on the body. It is important to study the influence of the choice of the communication protocol, the location of the device regarding communication, the antenna placement and orientation, and whether it can be influenced depending on the user. 

According to [58], which describes a classification method for users according to their use of the smartphone, features extracted from Bluetooth and WLAN connections and the time spent connected had a high impact on the demographic classification. Nevertheless, they do not provide the raw data to check the patterns and extract conclusions related to gender about them. In any case, from these results, we can state that wireless usage has differences according to gender. The availability to connect to a network or devices with the appropriate protocol is a key issue to consider. Is the availability of these connections dependent on gender?

Finally, the morphophysiology of bodies affects wireless communications, so we need to consider this for the choice of the device location and also for the technology used. This topic is of special interest if we consider that devices are worn on the body and are therefore highly affected by its morphology. For all kinds of bodies, communication devices should be worn on the external part of the body, with arms and shoulders being the preferred options, as these areas avoid obstacles when establishing communication. Even if the body map depicted in the study [59] is similar for men and women, the authors stated that “All body mass compositions are unique. Outside of the general guidelines, wearable systems using wireless communication should be tested thoroughly on a variety of people, and in a variety of settings”. 

New advances in wearable designs include the body as a communication channel (on-body communication). To study how communication affects the body, the propagation medium, and therefore the body model, must be considered. The waves known as Norton waves or Creeping waves allow for a theoretical estimation of the communication behavior in specific on-body scenarios [60]. These models consider aspects such as body size, human muscle, skin, and fat tissues. The design of this type of network must take into account the differences from a physiological and anatomical point of view of the bodies to accurately model both the channel and the communication model.

Another thing to consider is the choice of the frequency band used for communications, with different absorption and penetrations for different bands [61]. That is also related to the mass and composition of the body, so further research on gender differences is required because usually, a young male is selected as the final user [62].

Regarding communication, apart from the frequency selected considering the context and the routines, the orientation and the placement of the device and the antenna should also be taken into account as they are dependent on the movements of the user [63] to obtain the best signal strength and communications metrics.

### 2.6. User Interface (UI)

The way of interacting with electronic devices is a topic that has attracted a lot of attention, also in the case of wearable devices. Among most of today’s wearable devices, the most common method of interaction is still the touchscreen. That is performed by including the screen in the devices themselves, such as smartwatches, or by using the wearable just as a sensing system and accessing the information or settings through a mobile application. 

Analyzing this fact from a gender perspective, we can note several things. In the case of using the smartphone to access the wearable device, as it is less common for women’s clothing to have smartphone-friendly pockets, it may be a better option for women that the touchscreen is included in the wearable itself and not dependent on taking out the smartphone. This situation also applies to those notification methods that use smartphone vibration, which can become useless if the phone is not touching the body. In the case of wearables, this is an opportunity to explore. 

Regarding wearable devices, authors in [64] analyze and provide data disaggregated by gender on their preferred options for different methods of interaction (through the wearable screen or the smartphone) of a sports wearable device, while walking or running. In general, interactions through the device itself are preferred over interactions through the smartphone for this context and, logically, interactions while running are more complex than walking in both devices, without distinction by gender. Differences are found in the enjoyment of use, which arise more in women. In any case, both the sample and the interaction cases evaluated should be increased to obtain more comprehensive results.

However, it is necessary to go a step further. In the case of wearable devices, unlike other systems (PCs, tablets, smartphones), the methods of interaction can and should be more creative, as the devices are embedded in commonly used garments/complements or are mounted directly on the body, they can use this feature as an advantage to create new methods of interaction depending on their location, availability or accessibility, and overcome obstacles such as the impossibility of including screens/keyboards in some cases. Moreover, taking into account that cell phones are more cumbersome for women to carry (due to their size and lack of pockets), this opens up a very interesting field for the development of wearables that can replace the cell phone and can be worn on the body or embedded in the clothing itself [65].

Following this line, different forms of interaction have been proposed for wearable devices: gestural, haptic, or audio. For example, in the area of wearables for sports practice, the review presented in [66] concludes that in most cases, for user interaction, the devices deliver the information through visual interfaces (to improve cognitive knowledge or provide agglutinated information), and haptic and audio interfaces (when the aim is to improve specific physical goals while practicing sports).

Speaking of gesture-based interfaces, both gender and cultural background have a strong relationship with the predisposition of users to communicate through gestures [67]. In the rest of the cases, we have not found data disaggregated by gender that could confirm or disprove different interaction preferences by gender.

In the social context, interactions with devices are also influenced by the person’s location and activity. Thus, as stated in [68] (even this work only reports data related to smartphones), users’ attention to and acceptance of notifications is closely related to the location where they are and also to the activity being performed at the time. There are patterns of response behavior associated with different places and activities. Although this work does not provide data disaggregated by gender (it would be interesting to verify if these patterns differ), it is necessary to consider these profiles since daily activities are statistically different depending on gender (larger use of public transport versus private vehicles, more time dedicated to childcare and domestic tasks, higher proportion of part-time work, more prominent use of multitasking or less time focused on a single task, etc.).

As we can see, there is a need for a comprehensive study about preferred interaction methods for different types and styles of wearable devices, with different functionalities and performing different tasks, of course, with the results disaggregated by gender.

### 2.7. Location and Wearability

One of the most important decisions when designing a wearable is where on the body to wear the device. The location of the device and how comfortable and attractive it is to wear greatly influence the acceptance and use of the device, which is extremely important in devices specifically designed to be worn in public. In this aspect, we have to consider the comfort of the device, and this may be affected by texture, size, shape, weight, or temperature and should include the person’s usual movements.

When defining the location of a device, we must take into account how we are going to interact with it (as we have seen in the UI section), what magnitudes we need to measure and how (as explained in the sensors section) it influences communications, but also where users prefer to wear it. In this aspect, the wrist is usually the preferred place to wear it according to [69]. In fact, most of the wearables on the market today are watches or bracelets. In any case, although the study collects data, it does not disaggregate it by gender. According to the survey presented in [29], 95% of activity tracker wearables in the market are wrist-worn.

Regarding the location of the device, in [59], they make a very exhaustive analysis of where to place different wearable devices depending on their weight, the sensors used, communications, and the expected interaction with them. Similarly, the same author provides a web tool that, depending on the specifications entered for the wearable device, provides a body map of the most suitable areas for its location. It considers requirements in terms of size, form factor, discretion, interaction methods, sensors to use, movements to make, and communication technologies. It is also interesting to note that the map is given on two pictures, with masculine/feminine being different since there are factors to be taken into account depending on gender (in this case, solely based on anthropomorphic characteristics). 

When deciding the location of the wearable devices, the interrelation with sensors must be highly considered due to motion artefacts. This is important for every wearable device, but even more if the intended user is extremely probable to make big or fast movements as in the case of sports wearables. In [70], within the analysis of sports wearables, they analyze, among other characteristics, the wearability of the devices. Although not every work reviewed analyzes this aspect, it is important to note that the majority of those that do correspond to bracelets, and the location choice is related to the fact that this is a common place to wear watches or bracelets, and therefore, they are socially accepted. This relation to watches is interesting as this is one of the few accessories traditionally associated with the masculine. Outside the watch sphere, when it comes to sports devices, they are usually placed according to several reasons: the role of the device, the part of the body used in the sport or to be monitored, and the possibility of embedding an electronic device in a device already used during sports practice.

A fundamental part of wearability relies on checking how different devices and their location react to regular tasks. In this aspect, as we have seen in the sensors sections that some sensors as BVP are very impacted on by the activity of the user. Moreover, for UI, [64] analyze how devices behave and how users interact while walking or running. As we can see, device location and comfort are intimately related to user interaction. In any case, regular tasks, as we have stated before, differ by gender. Although changing this fact is a historical feminist claim, we cannot ignore it when designing wearables for different users, only being guided by activities ascribed to the masculine and ignoring those that men do not usually deal with. 

As for social factors that could influence, depending on gender, certain locations of the devices may be seen as obscene, even more, if it also involves direct interaction as being sexualized (for example, the chest area in the case of women) [67,71]. In general, women prefer to avoid attention to some areas of their bodies.

Part of the innovation may consist in seeing what tools or devices are already used on a regular basis (for a broader set of people) and making them intelligent (always to fulfill a demanded functionality, not just introducing technology) and not trying to embed computing and communications in a new device attached to your body.

### 2.8. Design, Aesthetics, Shape, Form Factor and Materials 

Finally, the design of the final device includes the form factor, material, shape, size and weight, and how visible it is.

Considering that gender is not something innate but is inscribed to a general framework of the social hierarchy of power distribution that continues to develop throughout life in collaboration and as a reaction/response to others, the design of the objects that surround us contribute to the definition of this framework (and can also serve to reverse it). Design stereotypes assigned to different genders have evolved throughout history. When designing inclusive devices, we must try to move away from these stereotypes, but it is also important to understand and acknowledge these biases and power structures for understanding how the devices will be perceived and used. When designing a device for a woman or a gender non-conforming person, the design must be based on them and their contexts, and also consider the consequences of this person as the final user, due to their gender.

Assuming designs that have been (and still are) conceived from androcentrism are gender-neutral is an error because they do not take into account the diversity of possible end users. On the other hand, it is also required not to fall into the stereotype when considering gender in design, and not take decisions based on what, in traditional terms, tends to be identified as masculinity and femininity and go beyond. In this regard, there are also cases of malpractice (changing the color, floral prints, or making them shiny, etc.), known as “pinking” [13].

The size of the device is a main issue and could sometimes be related to other factors such as the choice of the user interface, battery, or sensors and their proper functioning. For example, for GSR measurement, the size of the electrodes is closely related to the electronic circuit and therefore affects the quality of the sensing and final size of the device. Additionally, the size and shape can make the sensors not work properly: if the wristbands are too loose and do not fit (e.g., on small wrists), there can be problems in HR measurements, counting steps, or calculating activity level [6].

The type of closure chosen also has implications that can make it more uncomfortable for women [6] by causing it to get caught in the hair (usually longer) or sometimes thinner clothing. In this work, the authors analyze a device that, despite having a design conceptualized as feminine in terms of aesthetic preferences, has usability and fit problems since it follows the stereotypical premises of what could be liked more than actually making a useful device for the non-male public.

One of the fields in which we find more studies analyzing gender differences in the field of wearable devices is that of aesthetics possibly because it is more related to the area of the product design, fashion, and marketing. Thus, we found studies that assess the perceived value of the product in terms of aesthetics [72]. The results show a higher perception of the product’s utility linked to the device’s design for men based on aesthetics and not finding gender differences in terms of the perceived value in a hedonistic way, which at first, opposes gender stereotypes. Contrary to the collective imagination as well, in [73], the results indicate that men give more importance to the aesthetic aspect of the final product, contrary to women, who prefer a faster system response.

As for the shape and size, in bracelet wearables, women prefer smaller devices [8], and even in recent years, the trend tends to design unisex devices, women identify those called neutral designs with masculine characteristics: for example, “large, bulky with square screens and wide straps”, which suggests that although they are marketed as neutral, it is imbued with bias. That is why designers and engineers should pay attention and take a critical look at these seemingly neutral designs. Wearable devices should not be big and clunky if we want to design for everyone. On the other hand, it is also reported that men generally avoid using devices that are not intended for their gender. Ergonomics is important, and therefore designs seek more rounded devices. Further, ergonomics plays an important role in smartphones, where those of a smaller size tend to be more prevalent among women because they are easier to use (in general, the size of the screen is adapted to the size of the user’s hand—for using them with only one hand—being smaller in the case of women).

In addition to users’ personal preferences, there are other gender-related disadvantages related to the perceived women’s clothing or accessories, for example, as stated before, the lack of pockets in women’s clothing (or their small size, making them not functional) or the use of jewelry that can interfere with other wearables such as activity wristbands [74]. This feature can be, from the other perspective, an advantage for wearable device design, since, being used to wearing more accessories, adherence to wearable devices may be more prominent. It is also interesting to note, from an anthropological point of view, that wearables are more commonly integrated into watches, glasses or insoles, accessories used by men regularly, while there are few devices such as necklaces, earrings, rings, bras, bags or headbands, which are less common for men. This again confirms the androcentric bias in which a man is understood as a universal subject when thinking about design alternatives, limiting creativity and the plurality of solutions, thus discarding a large number of devices and applications.

According to [75], wearable devices defined as computational jewelry are designed for a feminine consumer, while those defined as technical gadgets are identified as gender neutral. One of the results of the study refers to the materials used for manufacturing these kinds of wearables, with silicon or plastic for those considered gadgets (unisex) and leather, silver, or metal for those referred to as jewelry. This conclusion could give a clue about the preferred materials used in these wearable accessories, but this study does not report a real assessment or data disaggregated by gender, so it is not possible to conclude if this reflects a gender stereotype or if these kinds of materials are the real preference.

Materials should also be considered if the wearable devices are integrated into clothes, as different fabrics are used for women’s/men’s clothing. Moreover, dress using a different number of layers is another factor to take into consideration. Finally, waterproofing is an attribute preferred by everybody, but should be taken into account depending on the final form factor and the typical tasks or routines of the final user (and if that depends on gender).

### 2.9. Conclusions

Once every aspect related to the electronic design of a wearable system is analyzed following a gender perspective, Table 2 summarizes the dependencies of the different blocks according to morpho-physiological factors, gender socialization, and those blocks where intersectionality (gender, racial, sexual, class, age, and functional ability diversity) should be considered.

## 3. Methodology Proposal

Once the factors that influence the design of the wearable device and the way they impact have been examined (it is recommended in any case to review the state of the art at the time of the design, since there may be new studies and conclusions that could improve the information from the previous sections), it is time to address the device design. 

To this end, it is convenient to have a methodology that incorporates and places the diversity of users at the center of the design. As we have said, this work addresses design from a gender perspective, but it would be interesting to broaden this framework with other diversities and add the intersectional approach. In any case, it is necessary to carry out a detailed analysis of the factors that influence every axis of the intersections to deepen our knowledge and achieve the design for all. 

In the literature, user-centered methodologies have been explored [76], but there are no established methodologies, rules, or guidelines that specifically include gender and its influence as a cornerstone. There is, however, a previous work that describes the design process of a specific wearable addressed from a gender perspective: the case of a monitoring device for cardiac activity [77]. This work addresses some of the specificities discussed in the previous sections, but with particular attention to the specific case of this final application. The final design is a bra that incorporates the electrodes and electronics needed to monitor cardiac activity for the requirements of wearability, being as discreet and comfortable as possible, and adapting to the female anatomy. Furthermore, in a similar way, but without focusing on the gender perspective, we have the Where to wear it project previously discussed [59]. This work focuses primarily on choosing the location of the wearable device once the requirements for functionality, data, size and communications are defined. 

Apart from these two works, there are no systematic guidelines for wearable device designs that consider the gender perspective to the best of our knowledge.

### 3.1. Interdependencies

One of the fundamental particularities extracted from the analysis section is that many of the wearable elements are related to each other, and therefore, the decision on the design of every block will influence the rest of them. Consequently, we cannot consider them as isolated blocks since, for example, the decision to use a screen in the wearable as a user interface will directly impact the size of the wearable, where to place it, and the processing and memory needed. In parallel, these new decisions will again have repercussions on other blocks, which in turn, once redesigned, will again influence others. 

That is why the design methodology will resemble a spiral, in which, if the solution to the problem is not approached systematically, we will be continuously redesigning each block and taking into account new interdependencies. 

**Figure 2:**
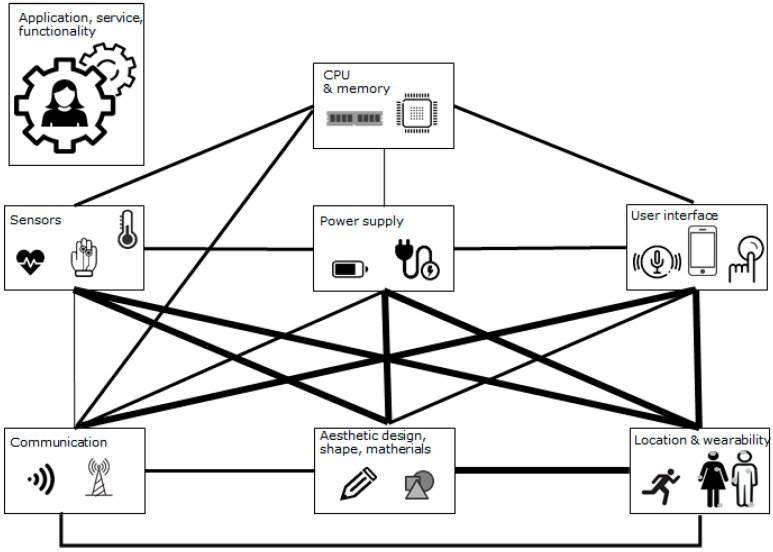
Interdependencies for wearable blocks.

Therefore, the first action must be to analyze the interdependencies so that they can be addressed as a whole. Figure 2 shows the relationships found between the different blocks where the thickness of the line connecting two blocks is proportional to the influence.

First and pervading the rest of the blocks is the general functionality of the system. Of course, the functionality and the purpose of the device is general, and affects and modulates the rest of the blocks. For the rest of them:

Sensors: the choice of sensors involving the variables to be monitored, the technology to be used, and the data collected influences almost all the blocks of the rest of the system. The choice of sensors and their measurement characteristics have a direct impact on the location of the device, since some variables can only be measured (or better measurements are obtained) in certain parts of the body (EMG of specific muscles, EEG, ECG, BVP, etc.). Additionally, the technology used can directly impact battery consumption. Indeed, all sensors must be powered, but some of them have very different energy demands (e.g., pulse monitoring by PPG includes the use of LEDs and photodetectors that increase power consumption). Likewise, signal processing and preprocessing depend on the technology used and the sensor itself (for example, the PPG signal is very noisy because of the technology itself, as mentioned above, and artifacts must be corrected; ECG and EEG signals are complex, requiring much more demanding processing to extract information). Depending on the characteristics of the sensed signal, the data rate can be very different (there are differences between an EEG signal and a temperature signal, which varies much more slowly). In addition, many wearable systems incorporate several sensors in order to enrich their capabilities, improve the features, and increase the information provided. Especially for areas such as activity monitoring, movement recognition or stimulation, or health state detection and diagnosis. Depending on the number of sensors and their nature (according to the variables required), it is possible to choose to incorporate all the sensors in a single device, or it may be necessary for the system to be composed of several devices (as the measurement points are far apart) in a way that is closely related to the location. In any case, the communications of the system will also be affected, both in the topology of the network to include several devices and in the characteristics of the transmission or quality of service required depending on the data to be transmitted. Finally, of course, the choice of sensors has an impact on the final design of the device, due to the size, materials and shape required to integrate the sensors.

Power supply: This part is a central block of the system on which all other modules depend. Indeed, the processing, sensors, user interface and communications have power requirements that will vary depending on the choice of each of the blocks. On the one hand, the choice of power supply (battery in most cases) has an impact on the final form factor of the device and is often the limiting factor for downsizing. On the other hand, if energy harvesting techniques are incorporated, the location of the device will be fundamental (usually piezoelectric takes advantage of the footstep when walking, for solar energy the device cannot be covered, or the location of the antenna for RF harvesting must be as unobstructed as possible).

CPU and memory: The choice of the memory and the processing block will be closely related, as discussed in Section 2, to the decision to process data on the edge or process it outside the device (fog/cloud computing). The communication network also influences this block, since its availability could require saving the data collected in the device while it is offline. As mentioned above, the choice of sensors will require more or less processing capacity, and, in the same way, the user interface will also determine the necessities in terms of computation power and memory (it is not the same in terms of the processing requirements for the use of buttons, touchscreen, or voice recognition as an interface, or even the use of the smartphone as an external interface, for example). Of course, the influence of complex data processing or even classification algorithms will also influence the device’s power consumption.

Communications: The choice of communications for the wearable will directly influence the rest of the system blocks, beginning with the mentioned power supply (communication protocols that involve more power consumption than others), following with the processing capacity and memory (depending on the availability of the network and the complexity of the protocol), and the choice of sensors (both for the amount of data to be transmitted and the potential existence of several connected devices because of the location of the sensors). In addition to these topics, the user interface and the communications used are closely related in every system that uses the smartphone as an external UI, since it will be indispensable to use a communications protocol available in the smartphone (usually Bluetooth or Wi-Fi, and to a lesser extent NFC). The use of wireless communications implies the location of the devices in the external area of the body as described in the communications section, and, if on-body communications are used to connect the different devices, it is necessary to model the communication channel between them as physiologically different parts of the body have distinct propagation characteristics. Finally, the encapsulation must permit communication with the electronics inside, avoiding fully hermetic metal encapsulations if these materials are used.

User interface: The UI is closely related to the location and the wearability of the device, as discussed in the previous section, because the interaction method must be accessible, comfortable, and easy to use in many circumstances (button manipulation, easily accessible keyboards or touchscreens, voice, etc.). Of course, the choice of the user interface will have a profound impact on the size, shape, weight, and appearance of the final device, being limited by it for usability reasons in some cases. In addition to these two blocks, the relationship between the choice of UI and the requirements in terms of power consumption, memory and processing capabilities have been described above, as well as the relationship between the chosen interface and the communications used. 

Design, shape and materials: Lastly, the product design in terms of shape, weight and materials, will be influenced by both the choice of sensors (type and technology used) and the user interface (buttons, displays). The type of power supply, recharging options and the use of energy harvesting techniques will also affect the final design (battery sizes, connectors, inclusion of solar panels, mass distribution for piezoelectric systems). The communication system used can modulate the use of materials and the final package design, both in common wireless and on-body communications. Finally, of course, in terms of size, shape, weight and materials, this block is closely related to the location of the device (those placed in clothing have a specific location, the weight determines in which parts of the body they can be worn) and its wearability (large and rigid devices are not suitable for hands, wrists, or joints, for example).

### 3.2. Methodology Proposal

Once the interdependencies between the different modules have been stated, we present a design methodology addressed from a gender perspective that allows the final design to move away from the androcentric vision.

For this purpose, it is important to apply the gender perspective throughout the whole project development cycle, from the very definition of the functionality to be provided, the diverse options to accomplish it, and the use of different methods. Furthermore, in the selection of every block of the system, it is necessary to examine what magnitudes are to be measured and how, the way the person will interact with the device and under what circumstances, where the device will be located, and how it affects the person in their daily life, how the system reacts to morphological and contextual differences, what are the implications of having such data, and whether biases are being replicated or biased data are being used at any stage of the process.

Furthermore, the validation tests on end-users should not be postponed until the final stages in the design cycle, but to include these users from the beginning, during the requirements definition. Therefore, not only the definition of the problem to solve, but also the functionality, the use cases, the context, etc., will benefit from a multidisciplinary and complete approach. 

#### 3.2.1. User Validation

When conducting validations with end users, in addition to having a varied and diverse sample, it is necessary to understand the social frameworks that operate according to gender (and others) when responding to people’s needs, taking into account their bases, circumstances, and the context. For that, it is crucial (and useful, in productivity terms) to use a qualitative methodology (often undervalued from the engineering, where there is a tendency to quantify everything) to use achievement metrics [78]. The use of a qualitative methodology is essential to understand the details of the system operation, the routines, the user needs, concerns, and power relations. In this way, we can have a greater general framework that helps provide better solutions. Of course, quantitative methodologies are also encouraged together with the qualitative approach.

Among the techniques we can use to integrate end-users into the design cycle (conception, selection and validation), we find different options such as brainstorming, in-depth interviews, focus groups, expert feedback, satisfaction tests, recording and visualization of use, and data collection from the device itself. Several of these options will be used throughout the proposed methodology.

#### 3.2.2. Methodology Description

**Figure 3:**
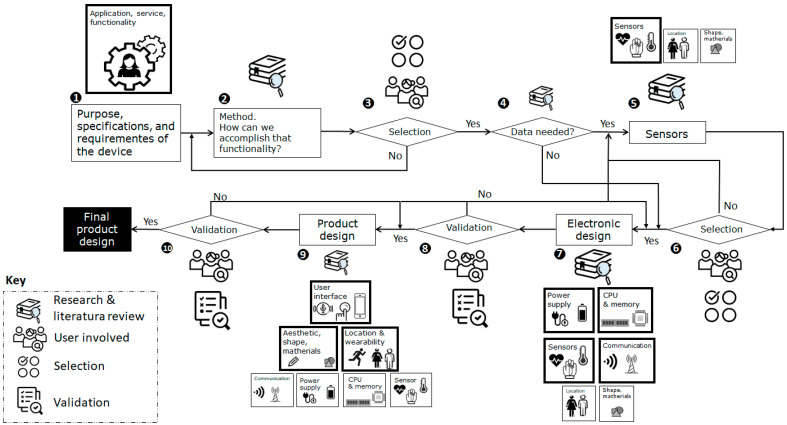
Proposed methodology. Thick boxes represent the module selection in the phase (number rounded in a black circle). Fine boxes are modules to consider because the interrelationships affect them.

When starting the design process of a wearable device, there are different factors to consider, and as we have seen, many of them are interrelated. This paper proposes the following methodology (shown in Figure 3) in order to simplify the design process and approach it systematically.

Due to the multidisciplinary approach required for the design and given that decisions made in any of the design stages can highly affect previous and subsequent decisions, it is necessary to evaluate and verify that every step is consistent with the design being carried out, the prior requirements and the needs, preferences and requests of the end-users, without forgetting, of course, the gender perspective.

The methodological proposal can be specified in the following stages, all of them addressed from a gender perspective.

**Stage 1. Definition of the functionality to be covered.**

Participants: End-users (must include women), experts (must include women) and the multidisciplinary design team (should include women).

This first stage must be approached considering a broad user group, as described in the analysis section. The aim of the wearable device or system must be defined and developed together with the end users. This group of users should be as mixed as possible and, in any case, women must be included in the group (both the end users and the designers/engineers). As we have seen, several areas are not covered yet, and the unmet needs of a large part of the population. 

This phase must undoubtedly involve end-users and for this, we can use techniques such as brainstorming or expert advice.

Once the final functionality is defined, it is also mandatory to assess the possible limitations, concerns, use cases and specific requirements that arise from applying a non-androcentric view. 

**Stage 2. Methods to fulfill the functionality.**

Participants: multidisciplinary design team.

Once the functionality is defined, options to implement it must be sought and thought of. Usually, the services or the functionality can be provided in different manners, and at this stage, based on the state of the art and the technique together with previous works, the possible options must be evaluated.

**Stage 3. Selection of the method.**

Participants: End-users, experts, and the multidisciplinary design team.

Once the different possibilities to fulfill the required functionality have been proposed, final users should evaluate them and discuss their preferences. This step is, together with the first one, essential to prevent undesired effects or usability problems of the designed devices. For this purpose, the team can use techniques such as in-depth interviews, focus groups, or preference and validation tests. It is important to include qualitative techniques at this stage. 

**Stage 4. Need to collect data.**

Participants: the multidisciplinary design team.

If the method to follow needs to collect data to implement the functionality, it is necessary to go to stage 5. If it is not necessary to sense data, go directly to step 7.

**Stage 5. Sensors.**

Participants: the multidisciplinary design team.

When approaching the design of a wearable device, one of the decisions that hardly influences the final design is the choice of sensors to use. As we have seen, it is necessary to evaluate the sensors to include based on which variables are most influential, the accuracy needed and its gender dependencies, which sensing technologies are most appropriate (in both cases taking into account sex and gender differences), and which ranges and deviations are influenced by gender. 

In making this decision, it is necessary to review the state of the art and count on data from previous work that includes women in the sample. If there are no previous data that include women in the sample for these variables/sensors, it is necessary at this stage to collect the necessary data to validate that the chosen sensors are accurate to fulfill the proposed functionality. 

The decision of which sensors to use can define or constrain the final form factor of the device, the material and its location. These two lateral interrelationships need to be considered, assessed and validated from a gender perspective.

**Stage 6. Selection of the sensors.**

Participants: End-users, experts and the multidisciplinary design team.

If there are different options for the sensors to use, final users should decide on the most convenient ones for them. If this is not the case, final users must validate the chosen sensors to collect the selected information. 

At this stage, it is also necessary to consider the privacy issues of the information collected and the possible malicious uses, with special attention on those derived from the existing gender inequalities usually not considered. Again, qualitative methods should be used for gathering the information and deepening the knowledge to anticipate future problems.

If the use of sensors is chosen/validated, proceed to step 7, otherwise, it is necessary to explore alternatives in step 6.

**Stage 7. Electronic design.**

Participants: the multidisciplinary design team.

Once the sensors to be used (if needed) have been defined, it is time for the system electronic design. This design will include the choice of the CPU and memory to use, the electronic design of the sensing circuits, the communications choice (and the specific protocol), and depending on all the above, the power supply of the system (and the inclusion or not of energy harvesting techniques). 

Throughout this process, the requirements defined in Stage 1 must be considered and fulfilled (with special attention to those derived from gender-specific situations, requirements and use cases), and the design team must validate that the device or system meets them. The battery duration, availability of communications, propagation differences, processing paradigm choice (edge/fog/cloud) and the appropriate energy harvesting techniques should be considered carefully due to women routines.

Again, decisions at this stage will influence the final shape and size of the device, the materials and the location of the wearable.

**Stage 8. Verification and validation of the electronic design.**

Participants: Experts and the multidisciplinary design team.

Once the proto device, or devices have been designed, they need to be validated by the end-users. Particularly susceptible are the availability of the communications if a standard protocol is used, the battery choice, its duration, and the charging method(s) according to the constraints reviewed in the analysis section. This validation step may be briefer than stage 6, since it is an intermediate design and will serve to detect problems that can be addressed before the final design moves forward. 

If the design is validated, it should be advanced to stage 9 to the final design, if not, it is necessary to go back to the conflictive point (stage 7 if the related to power supply or communications decisions or to stage 5 if the problems originate from the choice of the specific sensors or the technology used (inappropriate size or uncomfortable devices due to battery supply for the chosen area, excessive consumption of the sensors, etc.)).

**Stage 9. Product design.**

Participants: the multidisciplinary design team.

From the previous version of the electronic design, once validated, it is necessary to decide on the interaction methods and their implementation (taking into account the preferences of women in their choice) and once defined, to design the encapsulation and its final location (as well as whether it should be embedded in clothing or accessories).

It is necessary to especially consider the size and location requirements (especially those related to daily activities carried out by a variety of users, location, weight and size preferences, and suitability to the morphology and anatomy of women) collected in the initial definition, as well as in the rest of the stages where there has been feedback from the final users. 

Decisions at this stage could lightly modify the previous electronic design, but keeping requirements in mind. 

**Stage 10. Validation of the wearable.**

Participants: End-users, experts and the multidisciplinary design team.

This validation stage is the most comprehensive and complete, since it must cover the final device. 

When designing the test and validation phase, we can follow different approaches and perform quantitative and/or qualitative validation. As this phase includes the final validation of the system, both are included in this methodology. We can find in the literature examples of validation proposals for specific devices [79] that include guidelines to develop every validation phase. From the selection of the subjects to the measurements to be performed and the data to be collected. It is important to make a detailed test plan and that this plan incorporates a gender perspective in terms of a variety of subjects, tasks, routines and preferences. Regarding metrological validation, [19] provide as a conclusion of the study a series of recommendations for testing devices, among which we would like to highlight the emphasis on the specification of end-users in terms of gender “*test population should be described in terms of gender, age, weight, and height (or BMI), skin tone, etc*.”.

For this purpose, two types of validations are included. Of course, in both of them, women, as diverse as possible in terms of characteristics, social group, needs and usual routines, should be involved.

First, the functional validation of the device is important, including testing the definitive device by the final users and collecting usage data. To do this, data on the device itself should be gathered and if possible, the usage (through tests in a controlled space) and in a real-life case recorded. This stage will serve to technically validate that the data are correctly collected and processed, that the functionality is provided and that the device meets the functional requirements in a semi-controlled environment. At this stage, a quantitative evaluation method is mostly used.

On the other hand, in-depth interviews and end-user satisfaction tests will be conducted to check how users perceive, use and evaluate the wearable. At this stage, a qualitative evaluation will be mainly used.

If the design is validated, the final wearable will be ready to be released, if not, it is required to return to the redesign stage, not validated (stage 9 for UI, wearability, size, or aesthetic problems of the final product, stage 7 if new implementations need to redefine the CPU, power supply or communications methods, or stage 5 if the final have problems regarding the sensing stage).

With this methodological proposal, wearable electronic devices will be more applicable to different users, considering women and gender non-conforming people, and being more accurate in their purpose, creating more adherence to the user and reaching a wider population.

## 4. Use Case: Bindi 

To illustrate the proposed methodology in a real device design cycle, we are going to present the use case of Bindi, a wearable device for helping against gender-based violence. This section describes the motivation of the system, the requirements, the people involved in the design, and the methodology application for this case. 

### 4.1. Motivation

According to data from the World Health Organization (WHO), “violence against women (particularly intimate-partner violence and sexual violence) is a major public health problem and a violation of women’s human rights” [80] About 30% of women worldwide suffer from physical or sexual violence in their lifetime, mostly by their partners or ex-partners. In Spain, 1196 women have been murdered since 2003, the victims of gender-based violence [81]. It is essential to address this problem, which on the other hand, can be preventable if tackled from all areas of society, in a multisectoral long-term collaboration, and especially from public institutions through research, education, public policies, social services, economics, media and appropriate legislation. 

Gender-based violence elimination is the framework for the use case presented below. The aim is to develop a wearable system (named BINDI) that helps in the identification of risk situations, and provides assistance in these situations. 

### 4.2. People 

The team is an academic multidisciplinary group (UC3M4Safety) from the Universidad Carlos III de Madrid (Spain). The actors of the project are described below:

Research/Design Team: The group is an academic multidisciplinary team (UC3M4Safety) composed of researchers from areas such as electronics, telematics, computer science, sound/image processing and telecommunications engineers, together with psychologists, sociologists, gender studies specialists, journalists and lawyers who work jointly to achieve the purpose. The team is diverse (70% women and 30% men as a whole and 50% women/men in the specific engineering team), thus fulfilling the requirement of including women in the work/design team.

Experts: For the stages of the requirements definition, selection and validation, some professional experts (lawyers, social workers, psychologists, etc.) were consulted and included in the project. These experts, who work together with the victims of gender-based violence, provide first-hand information on functionality, usability requirements, and device design, as well as the specific characteristics of the final users. This information is crucial to define the final functionality of the device from a gender perspective.

Final users: Regarding the validation phases, a group of final users is included also in the project. This group is composed of three different profiles: (1) women who are not the victims of gender-based violence, (2) women victims of gender-based violence who do not suffer from post-traumatic stress disorder (PTSD) (usually women survivors whose situations of violence have been treated by specialists and have been overcome), and (3) the victims of gender-based violence with PTSD (usually women survivors still in treatment).

### 4.3. Procedure

Applying the methodology, we start from the needs detected by a group of professionals working with gender-based victims, who, together with the academics of the Gender Studies Institute, proposed a general system for fighting against this type of violence. The proposed solution is comprehensive, including the modification of the assistive protocols and public policies, but for this work, we will focus on the design of the technological device. 

As explained before, considering the final users’ requirements in the device design is essential to achieve good acceptance. It is also paramount to include a gender perspective in the entire process of analysis, conception, development and integration of the system. The final users will be women, and the electronics system design lacks incorporating a gender perspective into the process. In addition, these women share some special characteristics that drive the design to be even more singular. Integrating their requirements is essential for their acceptance, proper functioning, and guaranteeing women’s safety.

For that purpose, we have included the final users’ and experts’ assessments in the design process. A series of interviews are proposed to help us to collect detected issues for previous existing solutions, ideas for future designs, and specific problems related to their acceptance. For this stage of analysis, the technique of semi-structured in-depth interviews with professionals who work with gender-based violence victims is used. The interviews were carried out between March and August 2019. The corpus of analysis is composed of 14 group interviews and 20 individual ones, with a total of 59 participants (N = 59). The experts interviewed belonged to the teams of the 18 Centers of Attention to Women Victims of Gender-Based Violence in the Community of Madrid, 6 Civil Society Organizations that work with gender-based violence victims, and the Family and Women Unit of the National Police. More than 300 verbatims have been extracted and analyzed, giving us the qualitative approach. In the subsequent sections, the depicted quotes (in quotation marks) are direct translations, since the interviews were carried out in Spanish. 

Moreover, validation tests were performed with final users to assess the functionality, reliability, usability and acceptance. The tests were performed between 2020 and 2022 in several phases (more details are given below, in the design section).

### 4.4. Wearable Design

The design process according to the proposed methodology is described below:

**Stage 1. Definition of the functionality to be covered.**

Application definition: apart from the expected general requirement that has been requested “help in the gender-based violence reduction and detection”, experts have been interviewed for more specific requirements and functionality to be covered. Furthermore, these interviews covered concerns about acceptance/rejection, location, wearability, security and aesthetic thoughts. 

Firstly, we summarize the answers regarding the functionality suggested by the experts and the way of using the BINDI device automatically or proactively. 

Related to the service to be provided, the first thought was to develop a wearable with a panic button functionality. The experts point to their thoughts about this and their concerns about the implementation of a panic button that women can activate directly in a risky situation. The decision-making capacity to activate an alarm in a risky situation is questioned. Then, experts dig into the possibility to implement a wearable that launches the alarm autonomously in a risky situation, identifying the scene and the emotional circumstances. Concerns about reliability are also aroused. On the one hand, false alarms could be triggered and the victim can cancel them, but on the other, the victim’s lack of control over their situation and emotions are emphasized, detaching them from what happens to them and their lives. 

However, it is also worthy to highlight the different visions of some professionals on the design and location of the BINDI device. For example, the device would not have to be something portable for the victim, but rather something to be installed anywhere a woman can access. In the same way, the device could be worn by the men, placing the responsibility on the man instead of the woman. 

**Stage 2. Methods to fulfill the functionality.**

The next topic to be addressed is how to be aware of the situation. For that, the research team, after considering the possibilities, introduce the option based on affective computing, of identifying the emotions associated with the risky situation and triggering the alarm. The emotions that need to be measured and recorded with the BINDI device are assessed and the user that has to wear the device is considered. On the one hand, in Spain, no one can be forced to wear a device if they do not want to, and even less so if there has not yet been a conviction. On the other hand, women could wear it voluntarily, but the problem is that the responsibility for protection falls back on them. We, therefore, propose the solution of designing a device to be worn voluntarily by women and therefore, the emotion to be monitored should be fear.

**Stage 3. Selection of the method.**

The proposed solution is discussed and validated by the experts. In the opinion of the experts, fear, anger and anxiety would be of interest in triggering the system because they are the woman’s emotional expressions in these situations. 

According to the professional’s perspective, this way it would be essential to control, measure and detect if a risk situation is going to occur or not. Related to the difficulty of being able to correctly distinguish real risk situations, the problem of probable false alarms arises, which leads to the debate on the questioning of the device’s autonomous decision-making capability. Furthermore, even if the attack is not happening, this information related to emotions could be useful for the victim’s recovery process. 

Concerns arise because each person can react differently to the same situation, even though there are common reactions to certain stimuli. Thus, the alternative technique of individualized training of the device is suggested. 

Finally, another functionality to include and also related to the data access and uses could be the capability to record and register scenarios for future evidence of the situation they experience in trials. 

The last part of this first assessment is the opinions on the acceptance or rejection of the future developed device by the women, according to the professionals, for trying to avoid future problems. According to the experts, that depends on the recovery process moment. A woman who recognizes herself as a victim is going to accept the device in a different way than a victim who is not yet aware of it. In the same way, the acceptance will not be the same for those women who continue to maintain a relationship with the abuser or even continue living with him. 

Another possible reason for rejection could be the feeling of bringing back memories of what happened when having the device in mind. The presence of the device can give a feeling of overwhelming to the victim and not allow her to move on. 

Due to the possibility of device rejection, some professionals cite some recommendations to improve acceptance. A series of measures aimed both at working a priori with the victims and at demonstrating the proper functioning of the device, thus achieving confidence in it. 

Taking into account these last pieces of advice, for the first approach, the system would be intended for women who are already recognized by the system as victims, not living with the aggressor and being in therapy for their recovery.

**Stages 4 and 5. Need to collect data and sensors.**

The proposal is to design a comprehensive system that incorporates a series of wearable devices that allow the identification of states of fear and panic in women who have been victims based on AI. This system detects the emotion based on different physiological sensors (pulse, sweating and temperature according to the literature) and a microphone to detect the auditory context and the voice. Based on the emotion detected, an alarm will be triggered to a group of people decided by the woman or to the police. The system also includes the possibility of recording and verifying evidence that can later be used in legal proceedings. According to the Spanish law, audio and conversations recording are allowed as long as the recording subject is an active part of the conversation or situation. The system processes the audio and acoustic environment and only saves it if there are indications of being in a risky situation, in order to use it in court, if needed.

Thus, the selection of the physiological sensors to be validated is PPG, GSR and temperature. For that purpose, the research team proposes to design a bracelet (similar to the smart bracelets already on the market). For the auditory context, the team proposes to design a pendant that incorporates the microphone. It is necessary to design an ad hoc device, since similar smart bracelet solutions available on the market only include a pulse sensor and no GSR, thus lacking sensing data for fear detection. Among those available that enclose a GSR sensor (e.g., empatica), the system is closed and does not allow for ad hoc software development on the device to change sensing parameters or report risk situations, in addition to the high price of the options that allow access to raw data.

Due to the lack of sufficient data to train the system taking into account the gender perspective (most available databases do not have data disaggregated by gender, enough women in the sample are not intended to fear, so data for fear in women is scarce), at this stage, it was necessary to create our own database. This dataset would be used for training the automatic fear detection system. The generated dataset contains both the emotional stimuli [82] selected, taking into account the gender perspective, and the physiological [83] and audio responses of 100 women together with the labelling of the emotion experienced. 

**Stage 6. Validation of the selected sensors.**

The proposed solution is assessed by the experts and the data intended to be collected is validated. When analyzing the results of the interviews, many of the responses are related to the aesthetics and the location of the device. 

For the implementation of the BINDI device to be effective in the future, it is necessary to reflect on its design and location to adapt it to the reality that it faces. 

Taking into account the recommendations of the experts interviewed, a small, discreet and comfortable design is recommended as an everyday design, not invasive or uncomfortable. It is also mandatory that it cannot be identifiable by others so that the user who wears it does not feel an invasion of her privacy. An identification of this type of device also carries an added stigma that would harm the person wearing it. 

The design is viewed among the experts in a very discreet way, not only because of the added stigma it may have, but also because of the difficulty and risk that the woman may be exposed to by the aggressor or abuser. Most of the professionals interviewed warn of the control behaviors of the abusers toward the devices that the victim may have. 

For example, control over the mobile phone stands out, which is another control form. This could prevent it from triggering the alarm. These answers would be important also for the communication phase of the design. 

On the other hand, there are other aspects to take into account about the design of the device, referred to by some professionals, including avoiding damage to the device through permeability and taking into account wearability and comfort, so as not to have to remove it for daily activities, since it is believed that the device should be worn anytime because risk situations are not foreseeable. 

**Stages 7 and 9. Electronic design and product design.**

Once the sensors to be used have been defined and validated, the electronics design is approached. The electronics design is composed of two devices: a bracelet and a pendant. Both are based on the Nordic nRF52840 SoC that includes an ARM Cortex-M4 microcontroller plus a BLE transceiver. A BVP sensor (MAX30101), a custom design GSR and a temperature sensor (MAX30208), is the selected physiological sensor implemented in the smart bracelet, and a microphone included in the pendant for the auditory input. For power supply, we use the BQ2407xT and MAX17055, together with a rechargeable battery that lasts for 1 to 3 days depending on the user’s routines. Related to the edge/fog/cloud computing paradigm, we preprocess, clean and compress the signals in the wearable devices (edge). This information is sent to the user’s mobile phone, which serves as a gateway (that also include the functionality to cancel the alarm in the case it was a false alarm) to the Bindi server where the data are saved, processed and stored, encrypted to serve as digital evidence in an eventual trial.

The purpose of this work is to address the gender perspective methodology and not the design itself, so the device description is presented in [84] for further details. 

**Stage 8. Validation of the electronics design.**

The proposed electronics design is validated through two different approaches. First, using the information collected through the previous interviews with the experts working with gender-based violence victims. On the other hand, the specific decisions of the previous stage related to sensors, processor, communications, and power supply are validated by the multidisciplinary team including engineers, sociologists, psychologists and social workers. 

This decision has been made to reduce the number of iterations with the final users once we have the responses of the experts relevant to the electronics design. The final users’ evaluations are planned together with the validation tests for the final implementation, so for this phase, just the experts and the team participate.

**Stage 10. Validation of the wearable.**

This validation stage has been performed for the first time in Madrid with 15 women that were non-gender-violence victims in July 2022. For this first phase of testing, 15 women wore the devices for about 7–10 days during their normal daily lives, during which their physiological variables were monitored and data from the acoustic environment were analyzed. In addition, the volunteers were able to label the emotions they were experiencing at any given moment, either proactively or reactively. 

This first trial has been intended to provide feedback about the functionality and fix the problems of the devices. In these tests, three fundamental problems were found. The pulse measurements are not very accurate due to the user’s movement. On the other hand, the encapsulation of the bracelet, which was supposed to be waterproof, allowed sweat to seep through, causing the devices to fail. Finally, connection problems were also noticed, since, depending on the user’s routines (and correlating with her age), her phone was sometimes far away from the person and, therefore, it was not possible to establish a connection to upload the data. 

Based on the conclusions obtained, the wearable device is currently being redesigned for a new version of the bracelet that solves the problems of waterproofing and pulse measurement, and the inclusion of connection mechanisms that do not depend on the smartphone (NB-IoT, LTE, or LoRA) is being considered for the next version. 

A second phase of testing and validation with final users is planned for September 2023, with 20 participants (5 non-GBV victims and 15 GBV victims under supervision).

Considering the application of the methodology, throughout the design process of the device, decisions have been made specifically motivated by the need to consider the gender perspective. Particularly, we can highlight the following: 

Emotion classification algorithms: taking into account that the available databases for emotion classification are biased and that the results of subsequent studies are not disaggregated by gender, we have captured and generated a specific database with more than 100 female volunteers of different age ranges that would allow us to study the relevance and optimization of variables/characteristics and optimal algorithms for the detection of fear in women. The training of fear detection algorithms should include final users.

Sensors: the literature review yielded results that pulse and GSR were widely used variables for fear detection. However, no gender-disaggregated studies were found in the literature to confirm or disprove the applicability of these statements in women, just as there were no data or conclusions about the most relevant features. Further research is needed on this topic to generalize these statements and understand the different physiological responses to fear. To this end, the database acquired during the design of this device includes other physiological variables (respiration, EMG, EEG, presence of catecholamines) that will help us delve deeper into the subject, and select the most useful data variables and sensor technologies. 

UI: one of the requirements of the interviewed experts was that the device should be autonomous and proactively trigger the alarm. That is because of the fight or flight response to fear situations which, in the case of women, recent studies found that more than 70% experienced situations of another possibility: freezing or immobilization. The gender perspective is necessary to understand that victims’ reactions are sometimes different.

Wearability: during the functional tests, we have detected differences in sweating and humidity that could be due to the menopause condition, or social behavior. 

Communications: the distance between the smartphone and the devices should be considered for solving connection problems due to the use of handbags for carrying the smartphone. 

Aesthetics, design and materials: most experts consulted during the interview phase expressed concern about the devices being visible or noticeable for two reasons: the first, because of the danger of being detected by the aggressor, and the second, because they were considered a source of re-victimization. For this reason, they had to go unnoticed and look as similar as possible to an accessory in common use (necklaces, earrings, bracelets).

## 5. Conclusions

In this work, we have approached the design of electronic devices from a gender perspective to emphasize and highlight the need to delve into this aspect to provide better devices. The implications of electronics design needs to be rethought and analyzed from different points of view, and addressing the subject of gender will influence more than 50% of the population. 

Our purpose is to motivate the next generation of designers to introduce a gender perspective into their design process and with this goal, we propose a methodology that includes gender holistically. It is also important for researchers in the area that their work related to the different modules includes gender as one of the variables to be studied in order to advance knowledge and apply it in future designs. Nowadays we do not have enough data to ensure that the wearable devices designed are suitable for every women or gender non-conforming people. 

For that, we have presented an analysis framework examining every block included in a wearable device, attending to their anatomical and morpho-physiological impact. However, gender is not only circumscribed to anatomical differences; gender socialization plays an important role in women’s and non-conforming gender people’s lives that should also be taken into consideration. 

The main contributions of this work can be summarized as follows:

- We have analyzed both the social and anatomical implications that should be considered when designing a wearable. This analysis has been made placing the users at the center, from the necessity definition, the application to implement, and the implications of the decision of every module of the wearable: sensors, processing unit and memory, power supply, communications, user interface, location, wearability, materials and form factor. This analysis also aspires to be a helpful instrument for assessing and revising a gender perspective in existing wearables to improve them for everybody, leading to better performance and a higher adherence.
- Together with the analysis, as the main contribution of this work, we present a methodology to consider a gender perspective in the design cycle to address the gender perspective systematically. For that purpose, we previously examined the dependencies and relations of every module to keep them in mind for the presented methodology.
- Finally, the methodology is validated through a use case. The proposed system is BINDI, a device for helping in the fight against gender-based violence. We follow the stages of the proposed methodology for the electronic design to improve the process, validating it and assessing every step with final users and experts working with gender-based violence victims.

People’s needs are different, and right now, the world of wearables should consider them. It is time to open up the possibilities, be more creative and pay attention to a more diverse audience.

## Figures and Tables

**Table 1:** Summary for gender-perspective influences for physiological signals.

Variable	Anatomic	Socialization
Temperature	Absolute values; response to sudden changes; menstrual cycle; hormone therapy; pregnancy	Time spent in outdoor and indoor locations; task distribution
Cardiac activity	Heart rate absolute value; heart rate variability; different features for extracting values; response to stress, exercise or emotional states	Response to stress, exercise, aggressions or emotional states; daily activities and movements
GSR	Absolute values; response to sudden changes; menstrual cycle; menopause	Time spent in outdoor and indoor locations; task distribution
EEG	Spectrum power for all frequency bands; spatial distribution	Response to stress, aggressions or emotional states
EMG	Muscle response; muscle contractions /distensions to different movements; extension amplitude; inter-muscular patterns	Emotional expression muscle activation; daily activities and movements
Respiration	Difficulties during panic attacks; menstrual cycle; hormone treatments; menopause	Difficulties during panic attacks; daily activities and movements

**Table 2:** Summary for gender-perspective influences.

Element	Morpho-Physiology	Gender Socialization	Intersectionality
**Functionality**	X	X	X
**Sensors**	X	X	X
Temperature	X	X	X
Heart Rate	X		X
GSR	X	X	X
ECG	X		X
EEG	X	X	X
EMG	X		X
Respiration	X		X
**CPU & memory**		X	X
**Power supply**		X	X
**Communications**	X	X	X
**User interface**		X	X
**Location**	X	X	X
**Wearability**	X	X	X
**Design**	X	X	X

## Data Availability

Datasets collected in the use case presented for sensor selection validation and the machine learning training stage are available here: https://edatos.consorciomadrono.es/dataverse/empatia.
